# Seasonal dynamics of the gut microbiota in *Apis mellifera ligustica*: a two-year longitudinal study

**DOI:** 10.3389/finsc.2026.1920906

**Published:** 2026-07-31

**Authors:** Massimo Iorizzo, Gianfranco Pannella, Mariantonietta Succi, Sonia Ganassi, Dalila Di Criscio, Cosimo Tedino, Gianluca Albanese, Antonio De Cristofaro

**Affiliations:** 1 Department of Agricultural, Environmental and Food Sciences, University of Molise, Campobasso, Italy; 2 Unit of Food Science and Nutrition, Departmental Faculty of Science and Bio-Technology, Campus Bio-Medico University of Rome, Rome, Italy

**Keywords:** 16S rRNA sequencing, apis mellifera ligustica, gut microbiota, longitudinal study, microbial ecology, seasonal dynamics

## Abstract

The honey bee gut microbiota plays a crucial role in host nutrition, immunity, and colony health, yet the relative influence of seasonal and colony-specific factors on its long-term dynamics remains incompletely understood. This study investigated temporal variation in the gut bacterial community of three *Apis mellifera ligustica* colonies maintained in the same apiary and monitored over two consecutive years (2022–2023). Worker bees were sampled during eight seasonal periods, and gut microbiota composition was characterized using 16S rRNA gene amplicon sequencing. Across all sampling periods, the microbiome was consistently dominated by the characteristic honey bee-associated genera *Gilliamella*, *Snodgrassella*, *Bartonella*, *Frischella*, *Commensalibacter*, and *Lactobacillus*, indicating the persistence of a conserved core bacterial community. Seasonal variation was primarily associated with changes in the relative abundance of dominant taxa rather than with major changes in community composition. In particular, *Gilliamella apicola* and *Snodgrassella alvi* exhibited complementary seasonal patterns, with *Gilliamella* reaching its highest abundance during autumn, particularly in autumn 2023, whereas *Snodgrassella* predominated during spring and winter. Alpha-diversity metrics (Observed OTUs, Chao1, Shannon, and Simpson indices) showed limited seasonal variation, whereas beta-diversity analyses detected significant differences in community composition among seasons. Principal Coordinates Analysis and PERMANOVA identified season as the factor most strongly associated with microbiome variation, while colony identity did not significantly influence bacterial community composition under the standardized experimental conditions adopted in this study. Overall, these findings show that the gut microbiome of *A. mellifera ligustica* maintains a conserved core bacterial community while exhibiting reproducible seasonal variation in the relative abundance of its dominant members. This study provides a longitudinal baseline for future investigations aimed at understanding the ecological mechanisms underlying seasonal microbiome dynamics and their relationship with honey bee biology and environmental change.

## 1 Introduction

Honey bees (*Apis mellifera*) possess a specialized and relatively simple gut microbiota, consisting of a limited number of highly conserved bacterial taxa, including *Gilliamella*, *Snodgrassella*, *Bifidobacterium*, *Bombilactobacillus*, and *Lactobacillus* (1–3). The gut microbiota is established shortly after adult emergence through social interactions among nestmates and contact with hive-associated microbial reservoirs (2, 4). Despite its low taxonomic complexity, this microbial community plays a crucial role in host physiology, contributing to the digestion of pollen-derived compounds, nutrient assimilation, immune regulation, protection against pathogens, and detoxification of dietary and environmental substances (5–9). Consequently, alterations in microbiota composition may affect both individual bee fitness and overall colony health. Experimental perturbations of the gut microbiota, including antibiotic exposure, have been shown to disrupt community structure and increase honey bee mortality, highlighting the importance of microbial homeostasis for host survival (10). The composition of the honey bee gut microbiota is influenced by multiple factors, including host age, genetics, diet, environmental conditions, geographic location, seasonality, pathogen pressure, and management practices (2, 11). Among these, nutrition appears to play a particularly important role in shaping gut microbial communities (12–14). Dietary resources and supplemental feeding strategies can influence both bacterial composition and host health, as demonstrated by changes in intestinal microbiota associated with different overwintering diets (15).

Seasonal fluctuations in temperature, forage availability, brood rearing activity, and overwintering conditions can shape gut microbial communities throughout the year (8, 16, 17). Despite these sources of variation, the honey bee gut microbiota is generally characterized by the persistence of a conserved core bacterial community across geographical regions and environmental conditions (1, 2). Understanding how seasonal and environmental factors interact with the conserved core microbiota remains a major challenge in honey bee microbiome research.

Several studies have reported seasonal shifts in the relative abundance of dominant bacterial taxa. In temperate environments, winter-associated microbiotas often show increased abundance of taxa such as *Bartonella*, *Commensalibacter*, and specific lactobacilli, whereas spring and summer communities are generally enriched in bacteria involved in the metabolism of fresh dietary resources, including *Gilliamella* and *Snodgrassella* (16, 18–21). These observations suggest that the gut microbiota responds dynamically to seasonal changes and may contribute to the physiological adaptation of colonies to different environmental conditions (22).

Despite growing interest in honey bee microbiome ecology, current knowledge of seasonal dynamics remains incomplete. Most studies have focused on single annual cycles, overwintering periods, or comparisons between specific seasons, providing limited information on the temporal consistency of microbial patterns (16–18, 23). Longitudinal studies combining repeated seasonal sampling over multiple consecutive years while simultaneously controlling for colony genetic background and management conditions remain relatively uncommon. Such experimental designs provide a valuable framework for disentangling the relative contributions of seasonal environmental variation and colony-related factors to honey bee gut microbiome dynamics. To address these questions, this study investigated the gut microbiota of three *A. m. ligustica* colonies maintained in the same apiary and monitored over two consecutive years. *A. m. ligustica*, commonly known as the Italian honey bee, is one of the most widely managed and economically important honey bee subspecies worldwide owing to its widespread use in apiculture and its recognized suitability for managed beekeeping systems (24, 25). Importantly, the study was conducted on colonies headed by sister queens and maintained under identical environmental and management conditions, thereby reducing an important source of host genetic variability and allowing a clearer assessment of seasonal influences on gut microbiome dynamics. Using 16S rRNA gene amplicon sequencing, we characterised seasonal changes in bacterial community composition and diversity and evaluated the extent of colony-specific variability. This longitudinal approach provides new insights into the relative contributions of seasonal and colony-specific factors to the temporal dynamics of the honey bee gut microbiota.

## 2 Material and method

### 2.1 Study site and sample collection

The study was conducted in an experimental apiary located in Castelpoto (41°08′42″ N, 14°42′14″ E; 280 m a.s.l.), in the Province of Benevento, Campania Region, Southern Italy. Three A. m. ligustica colonies headed by sister queens from the same breeding lineage were selected to reduce the potential influence of host genetic variability among colonies. The colonies were monitored over two consecutive years (2022–2023) under identical management conditions. To standardise nutritional inputs, all colonies received the same supplementary feeding regimen. During winter, each colony received a single administration of 2.5 kg of commercial fondant composed of sucrose, glucose, and fructose (Fondabee^®^, Beernem, Belgium). At the beginning of spring, colonies were additionally supplemented with a 50% (w/v) sucrose syrup administered weekly for four consecutive weeks.

Sampling was performed weekly throughout each season. At each sampling event, fifteen adult worker bees were collected from each colony and immediately transported to the laboratory under refrigerated conditions. Honey bees were euthanised on dry ice and dissected according to the protocol described by (26). The entire intestinal tract was aseptically removed from each worker and stored at −80 °C.

At the end of each season, gut samples collected from the weekly samplings were pooled to obtain a single Seasonal Sampling Unit (SSU) representative of the entire season for each colony. Consequently, one SSU was generated for each colony and season, allowing assessment of seasonal microbiota dynamics while minimising the influence of short-term fluctuations occurring within individual weeks.

### 2.2 Environmental data collection and climate characterization of the study area

Meteorological data were retrieved for the apiary location in Castelpoto, Benevento, Italy (41°08′42″ N, 14°42′14″ E) using the Open-Meteo Historical Weather API. Daily records of mean, minimum and maximum air temperature, mean relative humidity, and precipitation were extracted for each meteorological season in 2022 and 2023. Seasonal values were calculated as averages for temperature and relative humidity, and as cumulative totals for precipitation. This seasonal aggregation was chosen because each Seasonal Sampling Unit (SSU) consisted of worker bees collected weekly throughout the corresponding season and pooled before microbiome analysis.

### 2.3 DNA extraction, amplicon library preparation and sequencing

Total genomic DNA was extracted from each Seasonal Sampling Unit (SSU) using the Stool DNA Isolation Kit (Norgen Biotek Corp., Thorold, ON, Canada) according to the manufacturer’s instructions. The bacterial community was characterized by amplification of the V1–V3 hypervariable regions of the 16S rRNA gene using the primer pair 27F (5′-AGAGTTTGATCMTGGCTCAG-3′) and 534R (5′ ATTACCGCGGCTGCTGG-3′) (27, 28). PCR amplicons were purified, quantified, and normalized before library preparation. Equimolar amounts of purified amplicons were pooled to ensure comparable sequencing depth among samples.

Sequencing was conducted by Eurofins Genomics Europe Sequencing GmbH (Ebersberg, Germany) on an Illumina MiSeq platform using a paired-end 2 × 300 bp sequencing strategy.

### 2.4 Bioinformatic processing

Raw sequence data were processed using the standardized Eurofins Genomics microbiome analysis pipeline. Forward and reverse primer sequences were identified by exact matching (no mismatches allowed) and removed prior to downstream processing. Reads lacking recognizable primer sequences or containing ambiguous nucleotides were discarded, and sequences were subsequently filtered according to the expected amplicon length. Chimeric sequences were identified using the *de novo* UCHIME algorithm (29), as implemented in VSEARCH (30), and excluded from subsequent analyses.

The remaining high-quality reads were processed using Minimum Entropy Decomposition (MED) (31, 32), an entropy-based sequence partitioning approach that identifies biologically meaningful sequence clusters by exploiting informative nucleotide positions while minimizing the influence of sequencing errors and low-abundance noise. According to the Eurofins workflow, MED partitions reads on the basis of informative nucleotide positions, allowing sequence decomposition at single-nucleotide resolution while reducing the influence of random sequencing errors and low-abundance noise.

Representative sequences from each MED-derived Operational Taxonomic Unit (OTU) were taxonomically assigned using DC-MEGABLAST against the curated NCBI nucleotide reference database (release 2020-02-03; well-classified sequences only). Taxonomic assignments were based on the lowest common ancestor of the best-matching reference sequences, requiring a minimum sequence identity of 70% across at least 80% of the representative sequence.

The resulting OTU abundance table and taxonomic assignments were subsequently processed in QIIME version 1.9.1 (33). Bacterial abundances were normalized using lineage-specific 16S rRNA gene copy numbers with CopyRighter (34). Sequences assigned to non-bacterial taxa, including chloroplasts and mitochondria, were removed before downstream ecological and statistical analyses.

### 2.5 Statistical analysis

Alpha diversity was evaluated using Observed OTUs, Chao1 richness, Shannon diversity, and Simpson diversity indices. Differences in alpha diversity among seasons were assessed using the Kruskal–Wallis test, followed by Dunn’s *post hoc* test with Bonferroni correction for multiple comparisons. Differences in alpha diversity between years were evaluated using the Wilcoxon rank-sum test. Beta diversity was evaluated using Bray–Curtis dissimilarity matrices and visualized by Principal Coordinates Analysis (PCoA). Differences in community composition were assessed using permutational multivariate analysis of variance (PERMANOVA) with 999 permutations to evaluate the effects of season, year, colony. Homogeneity of multivariate dispersion (PERMDISP) was assessed using the same Bray–Curtis distance matrix to verify that significant PERMANOVA results reflected differences in community centroids rather than unequal within-group dispersion. All statistical analyses were performed in R (version 4.3.2) using the *vegan* package (version 2.7-1). Statistical significance was accepted at *p* < 0.05.

## 3 Results

### 3.1 Environmental conditions during the study period

To characterize the environmental context of the study, seasonal temperature, relative humidity, and precipitation data were collected for the apiary located in Castelpoto (Benevento, Southern Italy) during 2022 and 2023. Marked differences were observed among seasons and, to a lesser extent, between years (**Table 1**).

**Table 1:** Seasonal environmental conditions recorded at the study site (Castelpoto, Italy) during 2022–2023.

Year	Season	Mean temp. (°C)	Mean min temp. (°C)	Mean max temp. (°C)	Mean relative humidity (%)	Total precipitation (mm)
2022	Spring	12.0	7.1	17.5	71.8	193.3
2022	Summer	25.1	19.0	31.7	56.1	86.5
2022	Autumn	15.7	11.5	20.7	78.5	518.9
2022	Winter	7.7	4.2	11.7	82.5	298.1
2023	Spring	12.0	8.0	16.5	79.4	370.9
2023	Summer	23.4	17.7	29.6	67.1	129.8
2023	Autumn	17.5	13.0	22.8	74.5	395.0
2023	Winter	7.4	3.9	11.6	82.5	265.4

As expected, summer was the warmest season in both years, whereas winter was characterized by the lowest temperatures. Summer 2022 was notably warmer and drier than summer 2023, with a mean temperature of 25.1 °C and cumulative precipitation of only 86.5 mm, compared with 23.4 °C and 129.8 mm in summer 2023. Conversely, spring 2023 was substantially wetter than spring 2022, with cumulative rainfall almost doubling that recorded in the previous year (370.9 vs. 193.3 mm).

Among all seasonal periods, autumn 2022 exhibited the highest precipitation levels, reaching 518.9 mm, while winter seasons were characterized by the highest relative humidity values (>82%). Relative humidity showed a clear seasonal trend, with lower values during summer, particularly in 2022 (56.1%), and higher values during autumn and winter.

Overall, the two years encompassed a broad range of environmental conditions, including warmer and drier periods as well as cooler and wetter seasons. Such variability provided a suitable framework for investigating the influence of seasonal environmental fluctuations on the gut bacterial communities of honey bee workers.

### 3.2 Sequencing output and dataset overview

A total of 24 Seasonal Sampling Units (SSUs), representing three colonies monitored across four seasons over two consecutive years, were analyzed by 16S rRNA gene amplicon sequencing. Following quality filtering and removal of non-bacterial sequences, sequencing depth ranged from 10,963 to 22,569 reads per sample, with an average of 14,354 reads per sample across the 24 SSUs. The final dataset comprised 1,539 bacterial Operational Taxonomic Units (OTUs), which formed the basis for subsequent taxonomic characterization and diversity analyses of the honey bee gut microbiota. Complete sequencing statistics, sample metadata, alpha-diversity metrics, and taxonomic abundance tables from phylum to species level are provided in **Supplementary Dataset S1**.

### 3.3 Taxonomic composition of the honey bee gut microbiome

#### 3.3.1 Phylum-level composition

The gut bacterial communities of honey bee workers were dominated by a limited number of bacterial at the family level, the honey bee gut microbiome was dominated by Orbaceae, Neisseriaceae, Bartonellaceae, Lactobacillaceae, and Acetobacteraceae throughout the two-year study. Orbaceae and Neisseriaceae consistently represented the largest proportion of the bacterial community across all seasons and years and were primarily represented by the genera *Gilliamella* and *Snodgrassella*, respectively. Bartonellaceae was consistently detected across all Seasonal Sampling Units, while Lactobacillaceae and Acetobacteraceae constituted additional stable components of the core microbiota, mainly represented by *Lactobacillus* and *Commensalibacter*, respectively. Overall, family-level composition remained highly stable over time, whereas the relative abundances of individual families varied seasonally, reflecting the taxonomic shifts observed at the genus and species levels.

#### 3.3.2 Family-level composition

At the family level, the honey bee gut microbiome was dominated by Orbaceae, Neisseriaceae, Bartonellaceae, Lactobacillaceae, and Acetobacteraceae throughout the two-year study. Orbaceae and Neisseriaceae consistently represented the largest proportion of the bacterial community across all seasons and years and were primarily represented by the genera *Gilliamella* and *Snodgrassella*, respectively. Bartonellaceae was consistently detected across all Seasonal Sampling Units, whereas Lactobacillaceae and Acetobacteraceae constituted additional stable components of the core microbiota, mainly represented by *Lactobacillus* and *Commensalibacter*, respectively.

#### 3.3.3 Genus-level composition

**Figure 1:**
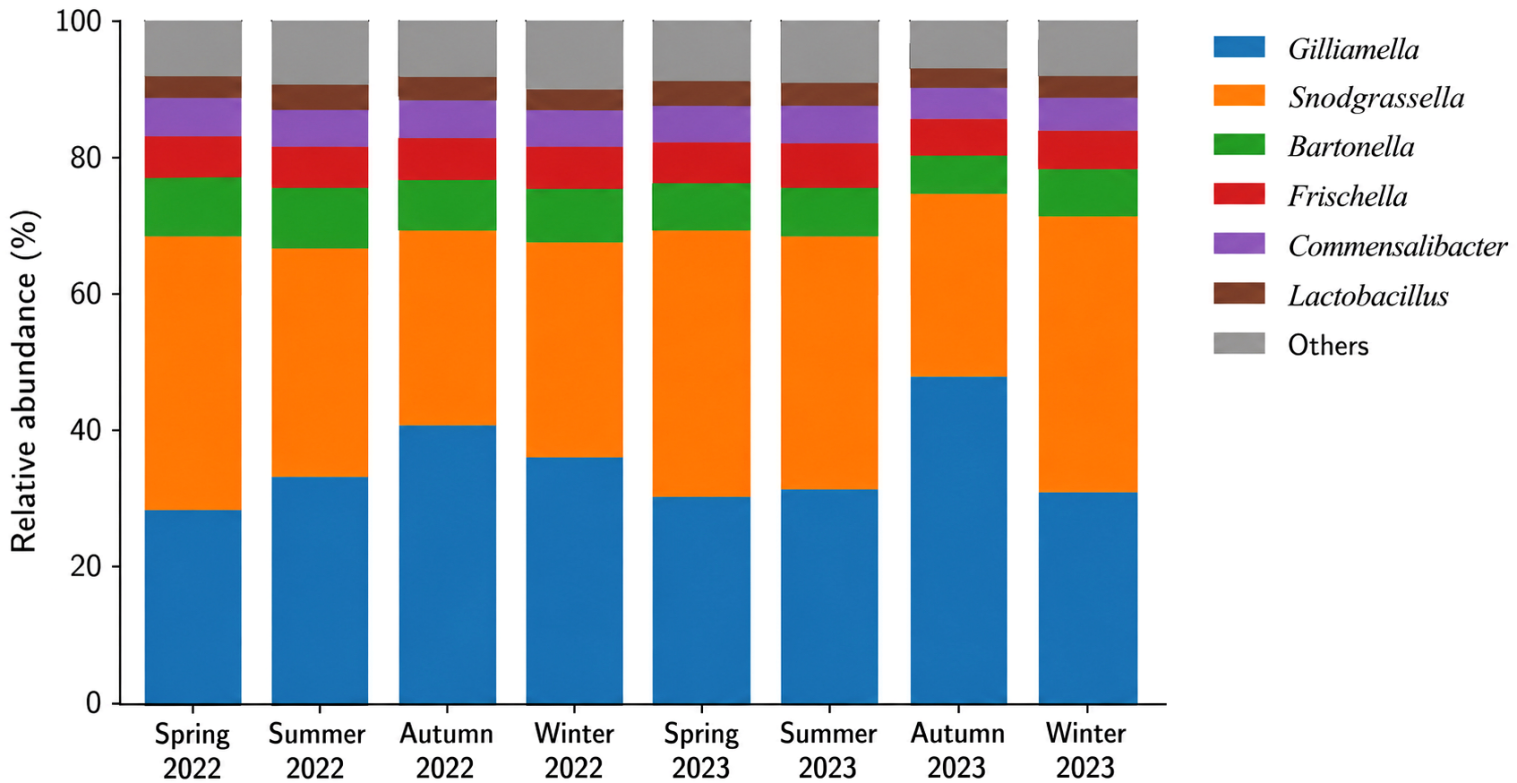
Seasonal taxonomic composition of the honey bee gut microbiome during 2022–2023. Stacked bar plots showing the relative abundance (%) of the dominant bacterial genera detected in worker bees from Colony 3, Colony 4, and Colony 6 across eight seasonal sampling periods. Low-abundance taxa were grouped into the category “Others”. Each bar represents one Seasonal Sampling Unit (n = 24 overall).

At the genus level, the honey bee gut microbiome was dominated by bacterial taxa typically associated with the honey bee gut ecosystem. The most abundant and recurrent genera were *Gilliamella*, *Snodgrassella*, *Bartonella*, *Frischella*, *Commensalibacter*, and *Lactobacillus*. Among these, *Gilliamella* and *Snodgrassella* were consistently detected in all Seasonal Sampling Units and represented the dominant members of the bacterial community. *Bartonella* was also consistently present across samples, whereas *Frischella* showed greater variation among colonies and sampling periods. *Commensalibacter* and *Lactobacillus* constituted additional stable components of the gut microbiota. Despite temporal fluctuations in their relative abundances, these dominant genera were consistently detected throughout the two-year study, indicating the persistence of a stable core bacterial community. The seasonal genus-level composition of the honey bee gut microbiome is shown in **Figure 1**.

### 3.4 Seasonal dynamics of the core honey bee gut microbiome

Seasonal averages were calculated from three independent colonies (n = 3) for each season and year.

Seasonal changes in the relative abundance of the dominant bacterial genera are shown in **Figure 1**. Although all core taxa were consistently detected throughout the study, marked seasonal fluctuations in their relative abundances were observed. Among the dominant genera, *Gilliamella* and *Snodgrassella* consistently represented the largest proportion of the bacterial community. *Snodgrassella* predominated during spring and winter, accounting for approximately 45–51% of the community, whereas *Gilliamella* generally increased during autumn. The most pronounced shift was observed in autumn 2023, when *Gilliamella* reached an average relative abundance of approximately 70%, becoming the predominant bacterial genus. At the family level, this pattern corresponded to an increase in Orbaceae and a concomitant decrease in Neisseriaceae.

Conversely, *Snodgrassella* showed the opposite trend, reaching its highest relative abundance during spring 2023 (50.7%) and winter 2023 (46.3%) before decreasing to 22.4% in autumn 2023.

The remaining core genera occurred at lower relative abundances but were consistently detected throughout the study. *Commensalibacter* reached its highest abundance during summer 2022 (10.1%) and remained detectable in all subsequent sampling periods. *Lactobacillus* showed its highest relative abundance during autumn 2022 (13.4%) and lower abundances during 2023. *Frischella* and *Bartonella* were also consistently detected across seasons, although their relative abundances varied among colonies and sampling periods.

### 3.5 Alpha diversity of the honey bee gut microbiome

**Figure 2:**
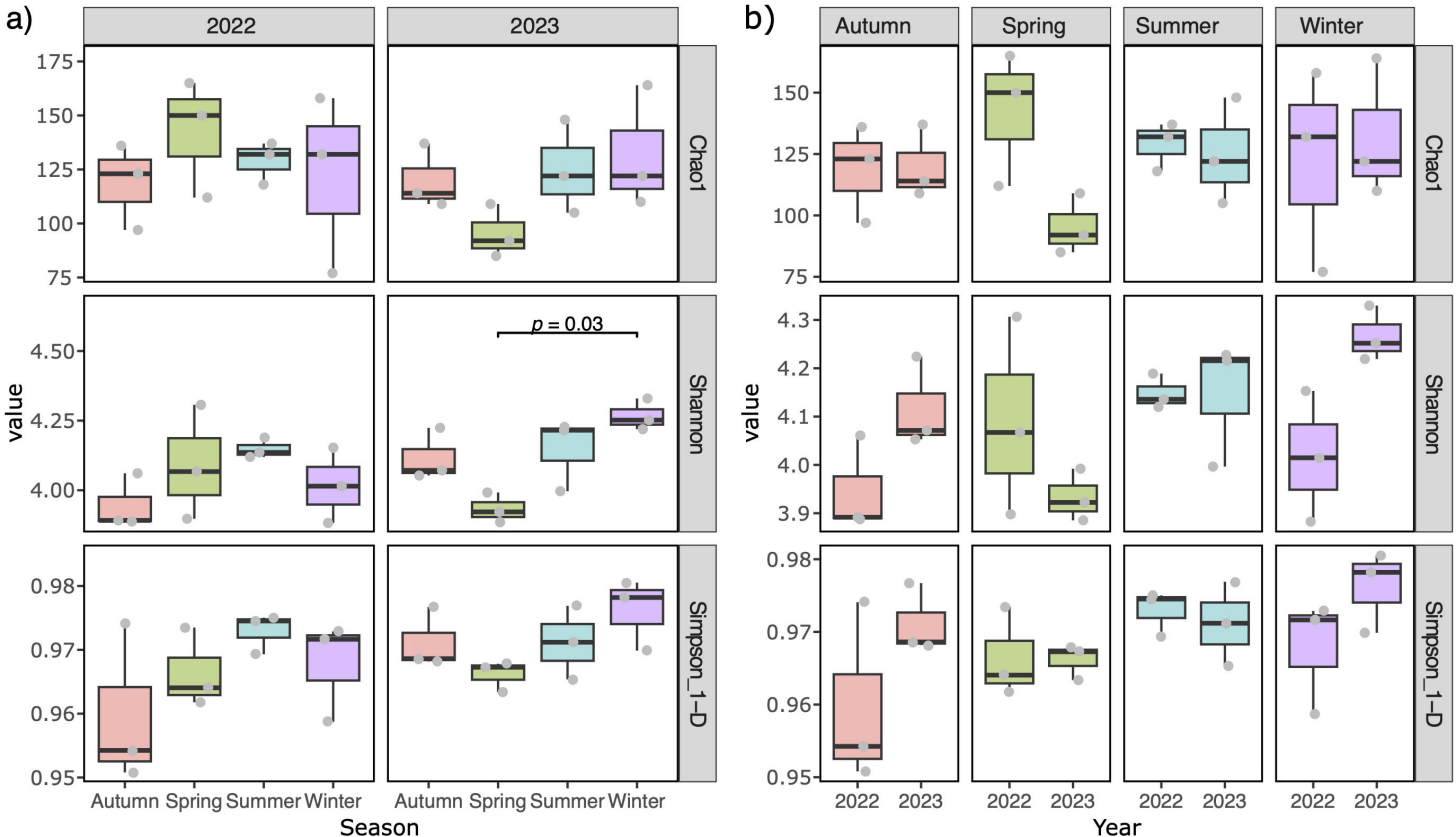
Alpha diversity of the honey bee gut microbiome across the **(a)** Season and **(b)** Year. Distribution of Chao1 index, Shannon and Simpson diversity indices calculated for the 24 Seasonal Sampling Units (SSUs) collected from Colony 3, Colony 4, and Colony 6 during 2022–2023. Each point represents one Seasonal Sampling Unit (n = 24).

Alpha diversity analyses were performed to evaluate seasonal and interannual variation in the richness and diversity of the honey bee gut microbiome (**Supplementary Table 1**). Diversity metrics were calculated from the complete dataset comprising 1,539 bacterial OTUs across 24 Seasonal Sampling Units (SSUs). Seasonal patterns in alpha diversity are presented in **Figure 2a**.

No significant seasonal differences (p > 0.05) were observed for Observed OTUs, Chao1 richness, or Simpson diversity in either 2022 or 2023. In contrast, Shannon diversity differed significantly among seasons in 2023 (Kruskal–Wallis test followed by Dunn’s *post hoc* test with Bonferroni correction, p < 0.05), with a significant difference between spring and winter.

Observed OTUs ranged from a mean of 95.3 in spring 2023 to 142.3 in spring 2022. Chao1 richness showed a similar distribution across seasons, with no significant seasonal differences detected in either year (**Figure 2a**).

Shannon diversity ranged from 3.93 to 4.27, reaching its highest value in winter 2023 (4.27 ± 0.06) and its lowest value in spring 2023 (3.93 ± 0.05). Simpson diversity remained consistently high throughout the study, ranging from 0.96 to 0.98, with no significant seasonal differences.

### 3.6 Beta diversity and community structure

**Figure 3:**
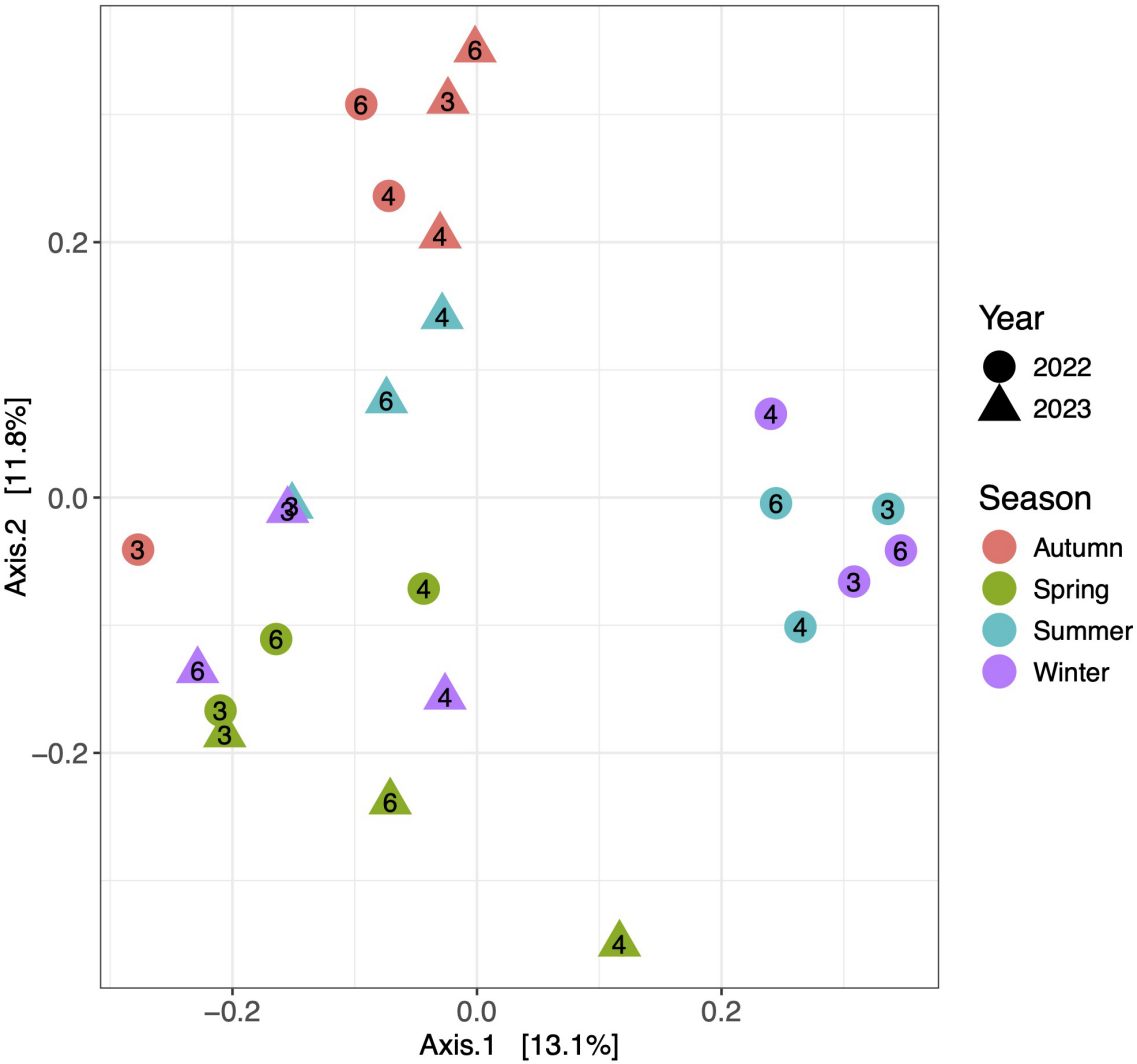
Bray–Curtis principal coordinates analysis (PCoA) of the honey bee gut microbiome across seasons and years. Ordination of the 24 Seasonal Sampling Units (SSUs) based on Bray–Curtis dissimilarity calculated from bacterial OTU relative abundances. Each point represents a sample collected from Colony 3, Colony 4, or Colony 6, and colors indicate sampling season. Seasonal means were calculated from three independent colonies (n = 3).

To investigate differences in the overall structure of the honey bee gut microbiome, beta diversity analyses were performed using Bray–Curtis dissimilarity metrics. Principal Coordinates Analysis (PCoA) showed a partial clustering of samples according to season (**Figure 3**; **Supplementary Dataset S3**).

The first two PCoA axes explained 13.2% and 12.2% of the total variation, respectively. Although some overlap among seasonal groups was observed, samples showed a greater tendency to cluster according to sampling season than according to colony identity.

The effects of season, year, and colony on bacterial community composition were evaluated by PERMANOVA (**Table 2**; **Supplementary Dataset S2**). Season significantly influenced microbiome composition (F = 1.77, R² = 0.210, p = 0.001), explaining approximately 21% of the observed variation. Year also had a significant but smaller effect (F = 1.51, R² = 0.064, p = 0.024), accounting for approximately 6% of the total variation. In contrast, colony identity did not significantly affect bacterial community composition (F = 0.84, R² = 0.074, p = 0.855). Together, season and year explained approximately 27.4% of the variation in community composition.

**Table 2:** PERMANOVA analysis of factors influencing honey bee gut microbiome composition.

Factor	df	F-value	R²	P-value
Season	3	1.77	0.210	0.001
Year	1	1.51	0.064	0.024
Colony	2	0.84	0.074	0.855

Analysis based on 24 Seasonal Sampling Units (SSUs) (n = 24).

Homogeneity of multivariate dispersion was evaluated using PERMDISP. No significant differences in multivariate dispersion were detected among seasons (F = 0.479, *p* = 0.730) or years (F = 1.360, *p* = 0.289). Therefore, the significant PERMANOVA results were not attributable to unequal within-group dispersion. Overall, beta-diversity analyses identified significant differences in bacterial community composition among seasons, whereas no significant effect of colony identity was detected.

### 3.7 Species-level drivers of seasonal variation

To further characterize the seasonal patterns observed at higher taxonomic levels, species-level profiles were examined across all sampling periods.

Species-level analysis identified *Gilliamella apicola* and *Snodgrassella alvi* as the most abundant bacterial species throughout the study. Their relative abundances varied across seasons, with the most pronounced change occurring in autumn 2023, when *G. apicola* became the predominant bacterial species. Conversely, *S. alvi* reached its highest relative abundances during spring and winter and decreased during autumn 2023.

Additional seasonal variation was observed for *Frischella perrara*, *Bartonella apis*, and members of the genus *Lactobacillus*, although these taxa occurred at substantially lower relative abundances than *G. apicola* and *S. alvi*.

**Figure 4:**
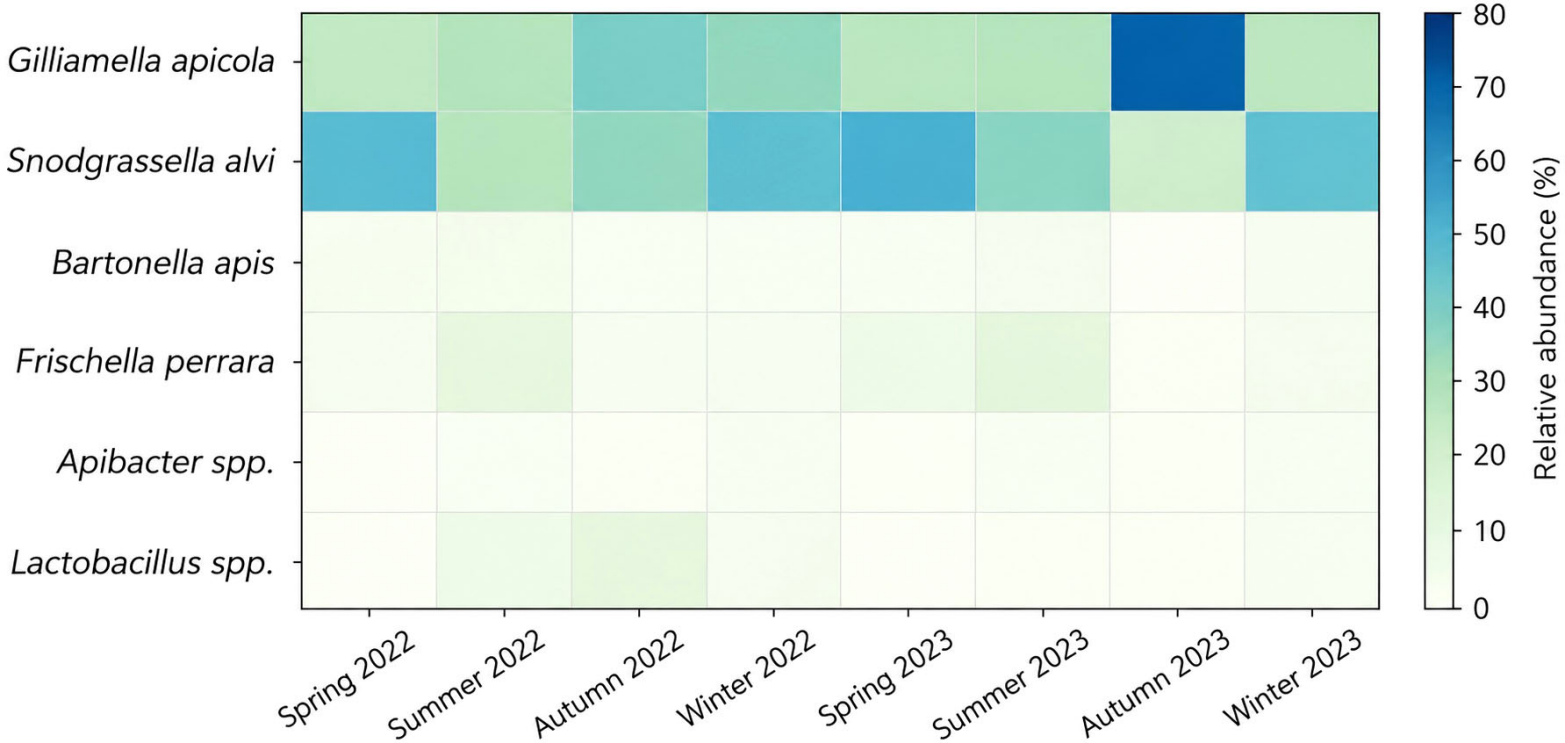
Heatmap of dominant bacterial species contributing to seasonal variation in the honey bee gut microbiome. Relative abundance patterns of the dominant bacterial taxa detected in worker bees from Colony 3, Colony 4, and Colony 6 across eight seasonal sampling periods (2022–2023). Color intensity reflects relative abundance. Seasonal means were calculated from three independent colonies (n = 3).

The seasonal distribution of the dominant bacterial species is illustrated in the heatmap shown in **Figure 4**.

## 4 Discussion

### 4.1 Stability of the honey bee core gut microbiome

The honey bee gut microbiome remained remarkably stable throughout the two-year monitoring period despite substantial seasonal variation in environmental conditions. Across all sampling periods, the bacterial community was consistently dominated by the characteristic honey bee-associated genera *Gilliamella*, *Snodgrassella*, *Bartonella*, *Frischella*, *Commensalibacter*, and *Lactobacillus*, confirming the persistence of a conserved core microbiome previously described in *A. mellifera* (1, 2, 9).

Our findings are consistent with previous studies reporting that seasonal and environmental variation primarily affects the relative abundance of dominant bacterial taxa while preserving the overall composition of the core microbiota (3, 16). Despite marked differences in temperature, humidity, and precipitation between seasons and years, the principal bacterial lineages remained detectable throughout the study. This observation is consistent with the hypothesis that the honey bee gut microbiome is maintained through continuous social transmission among nestmates and repeated exposure to colony-associated microbial reservoirs (2, 4, 23).

The relatively limited seasonal variation observed for Shannon, Simpson, Observed OTUs, and Chao1 indices further supports the temporal stability of the gut microbiome, despite seasonal shifts in the relative abundance of dominant taxa. Together with the beta-diversity analyses, these findings suggest that seasonal variation was associated primarily with changes in the relative abundance of resident bacterial taxa rather than with extensive changes in overall community composition (16, 18).

The limited variation observed in Shannon diversity across all samples further supports the remarkable Although the colonies experienced contrasting climatic conditions, including unusually dry summers and highly rainy autumns, these environmental variables were not analyzed quantitatively in relation to microbiome composition. Consequently, any ecological links between seasonal climatic conditions and microbiome restructuring should be interpreted with caution. Likewise, floral resource availability, nutritional intake, pathogen burden, and colony physiological status were not directly assessed and therefore cannot be considered causal drivers of the observed seasonal patterns.

Overall, the present results indicate that the honey bee gut microbiome combines long-term structural stability with seasonal compositional flexibility under the environmental conditions investigated. Future studies integrating environmental monitoring, dietary resources, colony health indicators, and functional microbiome analyses will be necessary to clarify the mechanisms underlying these seasonal dynamics.

### 4.2 Seasonal restructuring of the gut microbiome

Although the overall composition of the honey bee gut microbiome remained stable throughout the study, pronounced seasonal variation was observed in the relative abundance of several core bacterial taxa. The most evident changes involved *Gilliamella* and *Snodgrassella*, which consistently represented the dominant members of the bacterial community and exhibited reciprocal seasonal fluctuations across the two-year monitoring period. In particular, *Gilliamella* reached its highest relative abundance during autumn 2023, whereas *Snodgrassella* predominated during spring and winter. Similar seasonal patterns have previously been reported in honey bee populations from different geographic regions, although the magnitude and timing of these fluctuations vary according to environmental conditions and local ecological contexts (16–18, 21).

The complementary seasonal dynamics of these two dominant genera suggest that temporal microbiome variation was primarily associated with quantitative changes among resident core symbionts rather than with replacement of major community members. This interpretation is consistent with the beta-diversity analyses, which showed significant seasonal differences in community composition while alpha-diversity indices remained largely stable throughout the study period.

The functional characteristics of these bacterial taxa provide a possible ecological framework for interpreting the observed seasonal patterns. *Gilliamella* plays a central role in the degradation of complex plant-derived polysaccharides and pollen components that cannot be efficiently metabolised by the honey bee host alone (5, 6). Consequently, the increased abundance of *Gilliamella* observed during autumn is consistent with previous studies linking seasonal dietary variation and floral resource availability to changes in the honey bee gut microbiota (8, 15, 22). However, because floral resources, pollen composition, and nutritional intake were not directly quantified in the present study, this interpretation should be regarded as a plausible ecological hypothesis rather than a demonstrated causal relationship.

Conversely, *Snodgrassella alvi* is a specialized ileal symbiont that forms biofilm-like structures closely associated with the gut epithelium and has been implicated in gut homeostasis, immune stimulation, and maintenance of microbial community stability (2, 3). The higher abundance of *Snodgrassella* observed during spring and winter may therefore be consistent with seasonal changes in host physiology or nutritional status, although these variables were not directly evaluated in the present study.

Seasonal variation was also observed for *Frischella perrara*, *Commensalibacter*, and members of the genus *Lactobacillus*. Although these taxa occurred at substantially lower abundances than *Gilliamella* and *Snodgrassella*, their fluctuations contributed to the seasonal restructuring of the bacterial community. Similar patterns have previously been associated with seasonal changes in foraging activity, dietary composition, and hindgut metabolic processes (3, 8), although these factors were not specifically investigated here.

The seasonal clustering observed in the Bray–Curtis PCoA, together with the significant effect of season detected by PERMANOVA, indicates that seasonal differences were detectable at the whole-community level. In contrast, colony identity did not significantly influence microbiome composition, suggesting that under the standardized management conditions adopted in this study, seasonal variation exceeded colony-specific differences. Nevertheless, because the study included only three colonies from a single apiary, these findings should be interpreted within the context of the present experimental system and should not be generalized to other populations or environmental conditions without further validation.

Overall, the present results are consistent with a honey bee gut microbiome that maintains a conserved core bacterial community while exhibiting reproducible seasonal variation in the relative abundance of its dominant members. Under the environmental conditions investigated, seasonal dynamics were primarily characterized by quantitative shifts among resident bacterial symbionts rather than by extensive changes in community composition. Future studies integrating detailed environmental monitoring, floral resource characterization, colony physiological parameters, pathogen burden, and functional microbiome analyses will be necessary to clarify the mechanisms underlying these seasonal patterns.

### 4.3 Seasonality as a stronger driver than colony identity

One of the most notable findings of this study was that seasonal variation exerted a stronger influence on gut microbiome composition than colony identity. Although all three monitored colonies shared a highly similar core bacterial community, PERMANOVA identified season as the principal source of variation, whereas colony identity had no significant effect on bacterial community composition.

This observation differs from several previous studies reporting colony-specific differences in the honey bee gut microbiome, which have been associated with variation in queen genotype, colony health, management practices, nutritional resources, and local environmental conditions (2, 11, 23). However, many of those studies compared colonies maintained under different environmental conditions or in different geographic locations, making it difficult to distinguish colony-specific effects from broader ecological influences. In the present study, potential sources of colony-level variability were intentionally minimized by maintaining all colonies within the same apiary under identical management conditions and by using sister queens derived from the same genetic lineage. Under these experimental conditions, seasonal differences were more strongly associated with microbiome variation than colony identity.

The significant but comparatively smaller effect of year suggests that interannual environmental variability may also contribute to microbiome composition. Differences in temperature and precipitation between 2022 and 2023 could have influenced factors such as floral resource availability and foraging activity. However, because these ecological variables were not directly quantified, their contribution cannot be evaluated and any relationship should be considered speculative.

The seasonal clustering observed in the Bray–Curtis PCoA was consistent with the PERMANOVA results, with samples tending to group according to season rather than colony identity. A similar predominance of temporal and environmental effects over colony identity has also been reported by Rothman et al. (35), who observed that seasonal and ecological factors contributed more strongly to microbiome variation than colony-specific differences.

Nevertheless, these findings should be interpreted within the limitations of the experimental design. The study included only three colonies from a single apiary, and although this approach minimized genetic and management-related variability, it also limits the broader applicability of the results and does not allow colony, location, and environmental effects to be fully disentangled.

Previous studies have shown that neighboring honey bee colonies may differ in foraging preferences and resource exploitation (23, 36). Such differences were not evident in the present study, possibly because environmental and management conditions were highly standardized across colonies. Future investigations including multiple apiaries, larger numbers of colonies, and direct measurements of environmental and nutritional variables will be necessary to determine the relative contributions of season, colony identity, and local ecological conditions to honey bee gut microbiome dynamics.

Overall, our findings indicate that, under the controlled experimental conditions adopted here, seasonal variation was more strongly associated with microbiome composition than colony identity. These results provide further evidence that seasonal dynamics represent an important component of honey bee gut microbiome ecology while also highlighting the need for broader multi-apiary studies to evaluate the generality of these patterns.

### 4.4 Ecological significance, study limitations and future perspectives

The seasonal patterns observed in the present study provide further evidence that the honey bee gut microbiome represents a conserved yet dynamic microbial ecosystem. Although the overall taxonomic composition remained stable throughout the two-year monitoring period, marked seasonal variation was observed in the relative abundance of dominant bacterial taxa. These findings are consistent with previous studies indicating that the honey bee gut microbiome maintains a stable core community while exhibiting quantitative seasonal variation in its principal bacterial members (1, 2, 16).

The dominant bacterial taxa identified in this study are known to perform complementary metabolic and physiological functions that contribute to digestion, nutrient utilization, and gut homeostasis (2, 5, 6). Recent studies have further highlighted the importance of maintaining a stable gut microbiota for honey bee health and resilience under environmental stress (37, 38).

Consequently, the seasonal changes observed here may reflect ecological adjustments of the microbial community to changing environmental conditions. However, because floral resource availability, dietary composition, pathogen burden, and colony physiological status were not directly measured, these potential mechanisms cannot be demonstrated by the present data and should be regarded as plausible ecological hypotheses supported by previous literature rather than direct causal relationships.

Several limitations should therefore be considered when interpreting our findings. First, the study was conducted in a single apiary and included only three colonies. Although the use of sister queens and standardized management conditions minimized genetic and management-related variability, this experimental design limits the broader applicability of the results and does not allow colony, location, and environmental effects to be fully disentangled. Second, the study relied on 16S rRNA gene amplicon sequencing, which provides robust taxonomic characterization but limited functional information. Consequently, changes in bacterial relative abundance cannot be directly translated into changes in microbial metabolic activity. Third, members of the family *Bifidobacteriaceae*, frequently reported as characteristic components of the honey bee gut microbiota (39), were not recovered among the dominant taxa in the present dataset. This observation may reflect low relative abundance, primer- or region-associated bias related to V1–V3 16S rRNA sequencing, or limitations in taxonomic resolution, rather than a true ecological absence. Fourth, weekly samples were pooled within each season to generate a single Seasonal Sampling Unit (SSU). Although this strategy provided representative seasonal profiles while reducing short-term stochastic variation, it also reduced temporal resolution and may have masked finer fluctuations occurring within individual seasons.

Finally, although climatic variables were characterized, no direct measurements of floral resource availability, pollen diversity, nutritional intake, pathogen pressure, or colony physiological status were included. Consequently, the ecological drivers underlying the observed seasonal microbiome patterns remain to be experimentally verified.

Future investigations should therefore integrate broader spatial sampling with functional microbiome approaches. Studies including multiple apiaries, larger numbers of colonies, and different climatic regions will be essential to evaluate the generality of the seasonal patterns observed here. Likewise, combining metagenomics, metatranscriptomics, metabolomics, nutritional analyses, and colony health measurements will provide a more comprehensive understanding of the mechanisms linking seasonal environmental variation to microbiome composition and function (9). Long-term studies integrating microbiome dynamics with colony performance, disease resistance, and overwintering success will further clarify the ecological significance of the honey bee gut microbiome.

In conclusion, the present study indicates that the honey bee gut microbiome maintains a conserved core bacterial community while exhibiting reproducible seasonal variation in the relative abundance of its dominant members. Under the controlled experimental conditions investigated, season exerted a stronger influence on microbiome composition than colony identity. These findings contribute to the growing understanding of seasonal microbiome dynamics in honey bees while also highlighting the need for broader multi-site and functionally integrated studies to identify the ecological mechanisms underlying these temporal patterns.

## 5 Conclusions

This two-year longitudinal study showed that the honey bee gut microbiome maintained a conserved core bacterial community despite seasonal environmental variation. The dominant bacterial genera, including *Gilliamella*, *Snodgrassella*, *Bartonella*, *Frischella*, *Commensalibacter*, and *Lactobacillus*, remained consistently associated with worker bees throughout the study period, whereas seasonal variation was primarily reflected by changes in their relative abundance rather than by major changes in community composition. The use of genetically related *A. m. ligustica* colonies headed by sister queens and maintained under standardized management conditions minimized host genetic and management-related variability, allowing seasonal effects to be evaluated under controlled experimental conditions. Beta-diversity analyses identified season as the principal factor associated with microbiome variation, whereas colony identity did not significantly influence bacterial community composition within the experimental system investigated. Although the study was conducted in a single apiary and functional microbiome analyses were not performed, the present findings provide a valuable longitudinal baseline for understanding seasonal dynamics of the honey bee gut microbiome. Future multi-apiary studies integrating direct measurements of environmental and nutritional variables together with functional microbiome approaches (e.g., metagenomics, metatranscriptomics, and metabolomics) will be essential to clarify the ecological mechanisms underlying these seasonal patterns and to better understand the ecological mechanisms underlying seasonal microbiome dynamics and their relevance to honey bee biology and colony health.

## Data Availability

The datasets presented in this study can be found in online repositories. The names of the repository/repositories and accession number(s) can be found in the article/**Supplementary Material**.

## Supplementary material

**Supplementary Dataset S1** contains sequencing statistics, taxonomic assignments, OTU abundance data, Chao1 richness, and alpha-diversity metrics. **Supplementary Dataset S2** includes Bray–Curtis dissimilarity analyses, PCoA outputs, and PERMANOVA results. **Supplementary Dataset S3** reports the PCoA coordinates used for ordination analyses. **Supplementary Table S1** summarizes seasonal mean values of Observed OTUs, Shannon diversity, and Simpson diversity indices.
