# Supplementary material for: Seasonal dynamics of the gut microbiota in Apis mellifera ligustica: a two-year longitudinal study
Source: Front Insect Sci. 2026 Jul 31;6:1920906. doi: 10.3389/finsc.2026.1920906 (PMC13472841; doi:10.3389/finsc.2026.1920906)
Supplement: Supplementary file 1 [file DataSheet1.zip › supplementary material/Supplementary Data caption.docx]

**Supplementary Dataset S1.** Complete microbiome dataset and alpha-diversity metrics of the honey bee gut microbiome. The dataset includes sample metadata for the 24 Seasonal Sampling Units (SSUs), the complete Operational Taxonomic Unit (OTU) abundance matrix used for taxonomic analyses, alpha-diversity indices calculated for each sample (Observed OTUs, Chao1 richness, Shannon diversity, and Simpson diversity), and seasonal summary statistics. These data formed the basis for the taxonomic composition, alpha-diversity, and community structure analyses presented in the manuscript.
