# Supplementary material for: Seasonal dynamics of the gut microbiota in Apis mellifera ligustica: a two-year longitudinal study
Source: Front Insect Sci. 2026 Jul 31;6:1920906. doi: 10.3389/finsc.2026.1920906 (PMC13472841; doi:10.3389/finsc.2026.1920906)
Supplement: Supplementary file 1 [file DataSheet1.zip › supplementary material/Supplementary Table S1.docx]

**Table S1. Seasonal mean alpha-diversity indices of the honey bee gut microbiome**

| **Year** | **Season** | **Observed OTUs** | **Shannon** | **Simpson (1-D)** |
| --- | --- | --- | --- | --- |
| 2022 | Spring | 142.3 ± 27.3 | 4.09 ± 0.21 | 0.966 ± 0.006 |
| 2022 | Summer | 129.0 ± 9.8 | 4.15 ± 0.04 | 0.973 ± 0.003 |
| 2022 | Autumn | 118.7 ± 19.9 | 3.95 ± 0.10 | 0.960 ± 0.013 |
| 2022 | Winter | 122.3 ± 41.4 | 4.02 ± 0.14 | 0.968 ± 0.008 |
| 2023 | Spring | 95.3 ± 12.3 | 3.93 ± 0.05 | 0.966 ± 0.002 |
| 2023 | Summer | 125.0 ± 21.7 | 4.15 ± 0.13 | 0.971 ± 0.006 |
| 2023 | Autumn | 120.0 ± 14.9 | 4.12 ± 0.09 | 0.971 ± 0.005 |
| 2023 | Winter | 132.0 ± 28.4 | 4.27 ± 0.06 | 0.976 ± 0.006 |
